# Synthesis of new triazole-based trifluoromethyl scaffolds

**DOI:** 10.3762/bjoc.4.19

**Published:** 2008-05-29

**Authors:** Michela Martinelli, Thierry Milcent, Sandrine Ongeri, Benoit Crousse

**Affiliations:** 1 Laboratoire BioCIS-CNRS, Faculté de Pharmacie, Univ. Paris-Sud, rue J. B. Clément, F-92296 Châtenay-Malabry, France. Fax: +33 1 46 83 57 40.

**Keywords:** copper, catalysis, fluorine, heterocycle, click chemistry, peptidomimetics

## Abstract

Trifluoromethyl propargylamines react with various azide derivatives to afford 1,4-disubstituted 1,2,3-triazoles through a Huisgen 1,3-dipolar cycloaddition. The reaction is catalyzed by a Cu(I) species in acetonitrile, and the corresponding products are obtained in good yields. This process thus offers an entry to new trifluoromethyl peptidomimetics as interesting scaffolds.

## Background

The 1,2,3-triazole system has widespread uses, and it has been considered as an interesting component in terms of biological activity [1–5]. Although the use of heterocyclic moieties in peptidomimetics has been widely reported [6], the application of 1,2,3-triazoles in the field of conformational studies has occurred only recently [7–13]. In particular, Angelo and co-workers [8,12] reported the synthesis of triazole foldamers able to adopt specific protein-like conformations. On the other hand, it is well known that the introduction of fluorine atoms or a fluoroalkyl group can greatly modify the physico-chemical features and thus the biological properties of a molecule (resistance to metabolic oxidation and hydrolysis, modification of pKa, hydrophobicity,...) [14–17]. Furthermore, the development of CF_3_-containing scaffolds has gained a real interest especially in the peptidomimetic area [18–21]. In continuation of our interest in the synthesis of original trifluoromethyl compounds [22–26], and in order to study the influence of trifluoromethyl groups on the conformation of peptidomimetics, we decided to explore the preparation of trifluoromethyl triazole derivatives. Herein we turn our attention to the synthesis of new triazoles from trifluoromethyl propargylamines using the Huisgen 1,3-dipolar cycloaddition [27–29].

## Results and Discussion

**Scheme 1:**
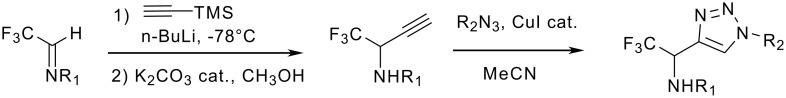
Synthetic approach to the trifluoromethyl triazoles.

The synthetic approach depicted in Scheme 1 shows that the desired compounds could be easily obtained via a 1,3-dipolar cycloaddition from the corresponding propargylamines which are obtained using an efficient procedure from the trifluoromethyl imines previously described by our group [30–32].

The copper(I)-catalyzed 1,3-dipolar cycloaddition [33–38] of organic azides and alkynes (also called “click chemistry”) resulting in the formation of 1,2,3-triazoles has become an increasingly attractive area [39]. According to the literature [33–38], the Cu(I) species can be used directly (*e.g.* CuI), or generated by oxidation of a Cu(0) or reduction of a Cu(II) species. Catalysis by the CuI is known to yield exclusively the 1,4-disubstituted regioisomer [33–34]. First, the N-(*p*-methoxyphenyl)-1-(trifluoromethyl)propargylamine was reacted with benzyl azide in the presence of CuI (10 mol%) and showed good reactivity with completion of the reaction within 24 h, whereas the use of CuSO_4_/Na ascorbate afforded the cycloadduct in low yield. The reaction was then carried out with different propargylamines (N-(p-methoxyphenyl) and N-benzyl) and various azides at room temperature in acetonitrile within 24 h which afforded the compounds **2a-i** with good yields (63-92%) after purification by column chromatography. The results are summarized in Table 1.

**Table 1:** Copper(I)-catalyzed synthesis of 1,4-disubstituted triazoles

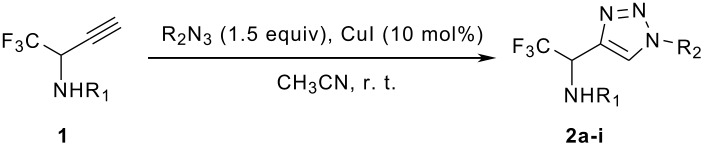				

Entry	R_1_	R_2_	Product	Yield (%)^b^

1	-PMP^a^	-Bn	**2a**	82
2	-PMP	-CH_2_COPh	**2b**	76
3	-PMP	-CH_2_OCOC(CH_3_)_3_	**2c**	73
4	-PMP	-CH_2_CO_2_CH_3_	**2d**	83
5	-PMP	-CH_2_CH_2_OH	**2e**	87
6	-Bn^a^	-Bn	**2f**	75
7	-Bn	-CH_2_COPh	**2g**	63
8	-Bn	-CH_2_CO_2_CH_3_	**2h**	92
9	-Bn	-(CH_2_)_2_OH	**2i**	73

^a^PMP: p-methoxyphenyl, Bn: benzyl. ^b^Yield after flash purification.

**Figure 1:**
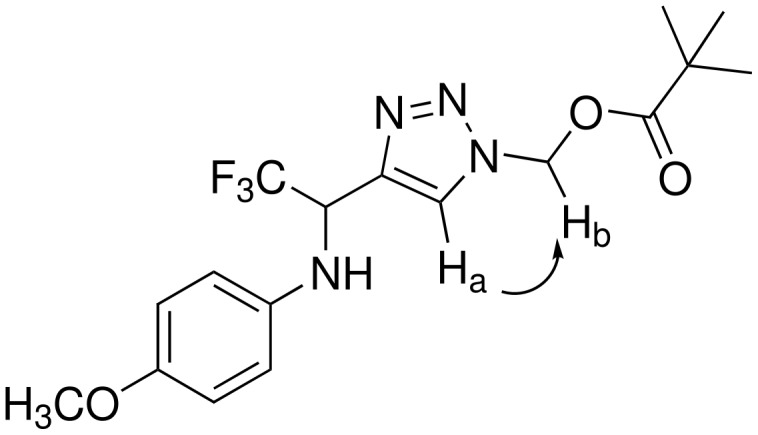
Experimentally found NOE correlation for compound **2c**.

As expected the new triazoles were formed in a fully regioselective manner affording the 1,4-regioisomer as highlighted from NOE experiments on compound **2c** (Figure 1). A strong correlation was observed between the hydrogen H_a_ and H_b_ respectively. The structure of the other compounds **2a-i** was assigned by analogy with **2c**.

**Scheme 2:**
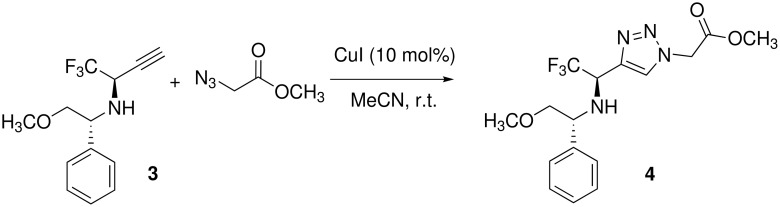
Cycloaddition of enantiopure propargylamine.

In our goal to study the influence of the CF_3_ group on the conformation of peptidomimetics, we applied our strategy to the enantiopure trifluoromethyl-propargylamine **3** bearing the removable (*R*)-phenylglycinol chiral auxiliary (Scheme 2) [30–32].

The reaction was carried out under the same condition with azidoacetic acid methyl ester and afforded the cycloadduct **4** in good yield (79%) and as a single isomer without any racemization. This compound can easily afford the free amino ester which is a promising trifluoromethyl building block for the synthesis of new triazole-based trifluoromethyl oligomers.

## Conclusion

In summary, this paper describes the synthesis of new trifluoromethyl triazole scaffolds from readily accessible propargylamines and azides through a copper (I) catalyzed 1,3-dipolar cycloaddition. The triazole derivatives were obtained in good yields and will be useful intermediates for further synthesis of new fluorinated foldamers and their conformational feature studies.

## Supporting Information

File 1 General methods, synthetic procedure and spectroscopic data of **2a-i** and **4**.
