# Supplementary material for: Synthesis of new triazole-based trifluoromethyl scaffolds
Source: Beilstein J Org Chem. 2008 May 29;4:19. doi: 10.3762/bjoc.4.19 (PMC2486457; doi:10.3762/bjoc.4.19)
Supplement: File 1 — General methods, synthetic procedure and spectroscopic data of 2a-i and 4. [file Beilstein_J_Org_Chem-04-19-s001.doc]

**Supporting Information**

**Synthesis of new triazole-based trifluoromethyl scaffolds**

Michela Martinelli, Thierry Milcent, Sandrine Ongeri and Benoit Crousse*

Address :Laboratoire BioCIS-CNRS, Faculté de Pharmacie, Univ. Paris-Sud, rue J. B. Clément, F-92296 Châtenay-Malabry, France. Fax: +33 1 46 83 57 40.

Email:Benoit Crousse* - [benoit.crousse@u-psud.fr](mailto:benoit.crousse@u-psud.fr); Michela Martinelli - [michela.martinelli1@virgilio.it](mailto:michela.martinelli1@virgilio.it); Thierry Milcent - [thierry.milcent@u-psud.fr](mailto:thierry.milcent@u-psud.fr); Sandrine Ongeri - [sandrine.ongeri@u-psud.fr](mailto:sandrine.ongeri@u-psud.fr)

*Corresponding author

Experimental Section

**General Methods**: Column chromatography was carried out on 254F Geduran SiO2 60 (40–63 μm). NMR spectra were recorded with a Bruker AC 200 or 300 spectrometer (^1^H: 200 or 300 MHz; ^19^F: 188 MHz; ^13^C: 75 MHz) in CDCl_3_ solutions. Chemical shifts are reported in ppm relative to Me_4_Si and CFCl_3_ [for ^19^F NMR (188 MHz, CDCl_3_, 25 °C)] as internal standards. For the ^13^C NMR data, reported signal multiplicities were measured relative to C–F coupling. The starting azides are obtained according to known procedures[[1]](#footnote-2).

**General Procedure for the Copper(I)-Catalyzed 1,4-Disubstituted 1,2,3-Triazole** :

To a solution of azide (0.5 mmol) and trifluoromethyl propargylamine (0.5 mmol) in acetonitrile (1.5 ml) was added a catalytic amount of CuI (10 mol%). Then the reaction was stirred at room temperature until completion (GC monitored). The solution was diluted with brine and the aqueous layer was extracted with AcOEt. The combined organic layers were dried (MgSO_4_) and filtered. After concentration under reduced pressure, the residue was purified by flash chromatography on silica gel.

**N-[1-(1-benzyl-1*H*-1,2,3-triazol-4-yl)-2,2,2-trifluoroethyl]-4-methoxybenzenamine (2a):**

Yellow oil, yield: 82%. ^1^H NMR (200 MHz, CDCl_3_) δ: 3.65 (s, 3H), 4.48 (d, ^3^*J* = 8.7 Hz, 1H), 4.95 (dq, ^3^*J* = 6.9, 8.6 Hz, 1H), 5.45 (AB system, ^3^*J* = 7.0, 8.6 Hz, 2H), 6.61 (m, 4H), 7.23 (m, 5H), 7.4 (s, 1H). ^13^C NMR (75 MHz, CDCl_3_) δ: 54.4, 55.2 (q, ^3^*J* = 32 Hz), 55.7, 114.9, 116.2, 122.0, 124.7 (q, ^3^*J* = 280 Hz CF_3_), 128.0, 128.9, 129.4, 134.1, 139.4, 141.6, 153.2.

^19^F NMR (188 MHz, CDCl_3_) δ: −75.6 (d, *J* = 6.9 Hz):

Elemental analysis for C_18_H_17_F_3_N_4_O: calculated: C, 59.66; H, 4.73; N, 15.46 found:C, 59.43; H, 4.52; N, 15.23

**2-{4-[1-(4-methoxyphenylamino)-2,2,2-trifluoroethyl]-1*H*-1,2,3-triazol-1-yl}-1-phenyl-ethanone (2b):**

Colorless oil, yield: 76%. ^1^H NMR (200 MHz, CDCl_3_) δ: 3.66 (s, 3H), 4.61 (d, ^3^*J* = 8.6 Hz, 1H), 4.99 (dq, ^3^*J* = 8.6, 6.8 Hz, 1H), 5.85 (m, 2H), 6.75 (m, 4H), 7.52 (m, 5H), 7.84 (s 1H,). ^13^C NMR (75 MHz, CDCl_3_) δ: 55.3 (q, ^3^*J* = 32 Hz), 55.5, 69.7, 114.9, 116.3, 121.3, 124.0, 124.8 (q, ^3^*J* = 282 Hz), 139.4, 141.6, 153.5, 177.8.

^19^F NMR (188 MHz, CDCl_3_) δ: −74.4 (d, *J* = 6.8 Hz)

Analysis for C_19_H_17_F_3_N_4_O_2_ calculated: C 58.46; H 4.39; N 14.35 found: C 58.23; H 4.32; N 14.51

**{4-[1-(4-methoxyphenylamino)-2,2,2-trifluoroethyl]-1*H*-1,2,3-triazol-1-yl}methyl pivalate (2c):**

Yellow oil, Yield: 73%. ^1^H NMR (200 MHz, CDCl_3_) δ: 1.16 (s, 9H), 3.67 (s, 3H), 4.48 (d, *^3^J* = 8.6 Hz, 1H), 5.09 (dq, ^3^*J* = 8.6, 6.9 Hz, 1H), 6.22 (s, 2H), 6.75 (m, 4H), 7.84 (s 1H,). ^13^C NMR (75 MHz, CDCl_3_) δ: 26.7, 38.8, 55.3 (q, ^3^*J* = 32 Hz), 55.5, 69.7, 114.9, 116.3, 124.0, 124.9 (q, ^3^*J* = 282 Hz), 139.4, 141, 153, 177.

^19^F NMR (188 MHz, CDCl_3_) δ: −75.4 ( d, ^3^*J* = 6.9 Hz)

Analysis for C_17_H_21_F_3_N_4_O_3_: calculated: C 52.85; H 5.48; N 14.50, found: C 52.80, H 5.62, N 14.52

**methyl 2-{4-[1-(4-methoxyphenylamino)-2,2,2-trifluoroethyl]-1*H*-1,2,3-triazol-1-yl}acetate (2d):**

Brown oil, yield: 83%. ^1^H NMR (200 MHz, CDCl_3_) δ: 3.66 (s, 3H), 3.77 (s, 3H), 4.49 (d, ^3^*J* = 8.6 Hz, 1H), 5.09 (dq, ^3^*J* = 8.6, 6.9 Hz, 1H), 5.15 (m, 2H), 6.75 (m, 4H), 7.74 (s 1H,). ^13^C NMR (75 MHz, CDCl_3_) δ: 51.2, 55.3 (q, ^3^*J* = 32 Hz), 55.5, 57.3, 114.6, 116.7, 123.0, 125.2 (q, ^3^*J* = 282 Hz), 139.3, 141.4, 153.6, 166.3.

^19^F NMR (188 MHz, CDCl_3_) δ: −75.4 (d, ^3^*J* = 6.9 Hz)

Analysis for C_14_H_15_F_3_N_4_O_3_ calculated: C 48.84; H 4.39; N, 16.27 found: C 48.92, H 4.53, N 16.23

**2-{4-[1-(4-methoxyphenylamino)-2,2,2-trifluoroethyl]-1*H*-1,2,3-triazol-1-yl}ethanol (2e):**

Yellow oil, yield: 87%. ^1^H NMR (300 MHz, CDCl_3_) δ: 3.65 (s, 3H), 4.10 (t, ^3^*J* = 6.4 Hz, 2H), 4.44 (t, ^3^*J* = 6.4 Hz, 2H), 4.48 (d, ^3^*J* = 8.5 Hz, 1H), 5.09 (dq, ^3^*J* = 8.5, 6.4 Hz, 1H), 6.65 (m, 4H), 7.84 (s 1H,). ^13^C NMR (75 MHz, CDCl_3_) δ: 52.7, 55.1 (q, ^3^*J* = 31.6 Hz), 55.7, 60.4, 114.9, 116.0, 124.0, 124.7 (q, ^3^*J* = 282 Hz), 139.4, 141, 153.6.

^19^F NMR (200 MHz, CDCl_3_) δ: −75.29 (d, ^3^*J* = 6.4 Hz)

Analysis for C_13_H_15_F_3_N_4_O_2_ calculated: C 49.37; H 4.78; N 17.71 found: C 49.12; H 4.56; N 17.86

**N-benzyl-1-(1-benzyl-1*H*-1,2,3-triazol-4-yl)-2,2,2-trifluoroethanamine (2f):**

Colorless oil, yield: 75%. ^1^H NMR (200 MHz, CDCl_3_) δ: 3.84 (d, ^3^*J* = 13.2 Hz, 1H), 3.91 (d, ^3^*J* = 12 Hz, 1H), 4.35 (q, ^3^*J* = 7.1 Hz, 1H), 5.42 (s, 2H), 7.25 (m, 10H), 7.41 (s, 1H). ^13^C NMR (75 MHz, CDCl_3_) δ: 51.6, 54.4, 56.3 (q, ^3^*J* = 30 Hz), 122.5, 124.7 (q, ^3^*J* = 290 Hz), 127.4, 128.1, 128.2, 128.5, 128.9, 129.5, 130.9, 138.9, 142.3.

^19^F NMR (188 MHz, CDCl_3_) δ: −74.9 (d, ^3^*J* = 7.1 Hz)

Analysis for C_18_H_17_F_3_N_4_ calculated: C 62.42; H 4.95; N 16.18 found: C 62.23, H 4.56, N 16.45

**2-{4-[1-(benzylamino)-2,2,2-trifluoroethyl]-1*H*-1,2,3-triazol-1-yl}-1-phenylethanone (2g):**

Oil, yield: 63%. ^1^H NMR (200 MHz, CDCl_3_) δ: 3.52 (d, ^3^*J* = 13.2 Hz, 1H), 3.81 (d, ^3^*J* = 13.2 Hz, 1H), 4.21 (q, ^3^*J* = 7.1 Hz, 1H), 5.75 (d, ^3^*J* = 18.5 Hz, 1H), 5.82 (d, ^3^*J* = 18.5 Hz, 1H), 7.25 (m, 5H), 7.41 (m, 5H), 7.52 (s, 1H). ^13^C NMR (75 MHz, CDCl_3_) δ: 51.2, 56.1, 56.4 (q, ^3^*J* = 31 Hz), 123.8, 124.8 (q, *^3^J* = 279 Hz), 127.3, 127.4, 127.5, 128.5, 128.7, 128.6, 137.2, 138.9, 141.6, 166.3.

^19^F NMR (188 MHz, CDCl_3_) δ: −74.9 (d, ^3^*J* = 7.1 Hz)

Analysis for C_19_H_17_F_3_N_4_O calculated: C 60.96; H 4.58; N 14.97 found: C 60.82; H 5.56; N 14.93

**methyl 2-{4-[1-(benzylamino)-2,2,2-trifluoroethyl]-1*H*-1,2,3-triazol-1-yl}acetate (2h):**

Yellow oil, yield 92% ^1^H NMR (200 MHz, CDCl_3_) δ: 3.21 (s, 3H), 3.83 (d, ^3^*J* = 13.2 Hz, 1H), 4.21 (q, ^3^*J* = 7.1 Hz, 1H), 4.23 (q, ^3^*J* = 7.1 Hz, 1H), 5.73 (d, ^3^*J* = 18.5 Hz, 1H), 5.82 (d, ^3^*J* = 18.5 Hz, 1H), 7.22 (m, 5H), 7.35 (s, 1H). ^13^C NMR (75 MHz, CDCl3) δ: 50.2, 50.8, 51.6, 56.3 (q, ^3^*J* = 30 Hz), 121.8, 124.8 (q, ^3^*J* = 279 Hz), 127.5, 128.5, 128.7, 138.9, 141.6, 168.3.

^19^F NMR (188 MHz, CDCl3) δ: −75.3 (d, ^3^*J* = 7.1 Hz)

Analysis for C_14_H_15_F_3_N_4_O_2_: calculated: C 51.22; H 4.61; N 17.07 found: C 51.32, H 4.52, N 17.12

g

**2-{4-[1-(benzylamino)-2,2,2-trifluoroethyl]-1*H*-1,2,3-triazol-1-yl}ethanol (2i):**

White powder, yield: 73%. ^1^H NMR (200 MHz, CDCl_3_) δ: 2.56 (s br., 2H), 3.8(d, ^3^*J* = 12.5 Hz, 1H) 3.81 (d, ^3^*J* = 12.5 Hz, 1H), 3.97 (t, ^3^*J* = 5 Hz, 2H), 4.22 (q, ^3^*J* = 7.3 Hz, 1H), 4.4 (t, ^3^*J* = 5 Hz, 2H), 7.25 (m, 5H), 7.62 (s, 1H). ^13^C NMR (75 MHz, CDCl_3_) δ: 51.5, 52.8, 56.2 (q, *J* = 30 Hz), 60.9, 123.9, 124.5 (q, J = 279 Hz), 127.5, 128.3, 128.6, 138.8, 141.8.

^19^F NMR (188 MHz, CDCl_3_) δ: −74.8 (d, *^3^J* = 7.3 Hz)

Analysis for C_13_H_15_F_3_N_4_O calculated: C 52.00; H 5.04; N 18.66 found: C 51.82; H 5.12; N 18.85

**methyl 2-(4-{(*R*)-1-[(*R*)-2-methoxy-1-phenylethylamino]-2,2,2-trifluoroethyl}-1*H*-1,2,3-triazol-1-yl)acetate (4):**

Yellow oil, yield: 79%. ^1^H NMR (200 MHz, CDCl_3_) δ: 3.22 (s, 3H), 3.28 (dd, ^3^*J* = 3.9, 9.6 Hz, 1H), 3.36 (t, ^3^*J* = 9.6 Hz, 1H), 3.71 (s, 3H), 3.85 (dd, ^3^*J* = 3.9, 9.6 Hz, 1H),4.12 (q, ^3^*J* = 7.5Hz, 1H), 5.11 (s, 2H), 7.25 (m, 5H), 7.63 (s, 1H). ^13^C NMR (75 MHz, CDCl_3_) δ: 50.8, 53.1, 53.9 (q, ^3^*J* = 30.7 Hz), 58.5, 59.1, 77.3, 124.2, 124.5 (q, ^3^*J* = 279 Hz), 127.9, 128.1, 128.7, 138.7, 142.5, 166.

^19^F NMR (188 MHz, CDCl_3_) δ: −75.0 (d, ^3^*J* = 7.5 Hz)

Analysis for C_16_H_19_F_3_N_4_O_3_ calculated: C 51.61; H 5.14; N 15.05 found: C 51.65; H 5.24; N 15.12

1. Loren J. C.; Krasinski, A.; Tokin, V. V.; Sharpless, K. B. *Synlett* 2**005,** *18,* 2847–2850. doi: 10.1055/s-2005-918944; Lu, X.; Bittman, R. J. *Org. Chem.* **2005,** *18,* 4746–4750. doi:10.1021/jo050513u [↑](#footnote-ref-2)
